# Interacting Effects of Light and Iron Availability on the Coupling of Photosynthetic Electron Transport and CO_2_-Assimilation in Marine Phytoplankton

**DOI:** 10.1371/journal.pone.0133235

**Published:** 2015-07-14

**Authors:** Nina Schuback, Christina Schallenberg, Carolyn Duckham, Maria T. Maldonado, Philippe D. Tortell

**Affiliations:** 1 Departement of Earth, Ocean and Atmospheric Sciences, University of British Columbia, Vancouver, BC, Canada; 2 School of Earth and Ocean Sciences, University of Victoria, Victoria, BC, Canada; 3 Department of Botany, University of British Columbia, Vancouver, BC, Canada; Mount Allison University, CANADA

## Abstract

Iron availability directly affects photosynthesis and limits phytoplankton growth over vast oceanic regions. For this reason, the availability of iron is a crucial variable to consider in the development of active chlorophyll a fluorescence based estimates of phytoplankton primary productivity. These bio-optical approaches require a conversion factor to derive ecologically-relevant rates of CO_2_-assimilation from estimates of electron transport in photosystem II. The required conversion factor varies significantly across phytoplankton taxa and environmental conditions, but little information is available on its response to iron limitation. In this study, we examine the role of iron limitation, and the interacting effects of iron and light availability, on the coupling of photosynthetic electron transport and CO_2_-assimilation in marine phytoplankton. Our results show that excess irradiance causes increased decoupling of carbon fixation and electron transport, particularly under iron limiting conditions. We observed that reaction center II specific rates of electron transport (ETR_RCII_, mol e- mol RCII^-1^ s^-1^) increased under iron limitation, and we propose a simple conceptual model for this observation. We also observed a strong correlation between the derived conversion factor and the expression of non-photochemical quenching. Utilizing a dataset from in situ phytoplankton assemblages across a coastal – oceanic transect in the Northeast subarctic Pacific, this relationship was used to predict ETR_RCII_: CO_2_-assimilation conversion factors and carbon-based primary productivity from FRRF data, without the need for any additional measurements.

## Introduction

The photosynthetic assimilation of inorganic CO_2_ into organic carbon by marine phytoplankton accounts for almost half of total global primary productivity [1], and variations in phytoplankton primary productivity can profoundly affect ecosystem dynamics and global climate (e.g. [2–5]). However, despite its recognized importance, it remains challenging to accurately quantify marine primary production at the temporal and spatial resolution needed to relate its variability back to external environmental conditions. In vast oceanic regions, the availability of iron (Fe) limits marine phytoplankton primary productivity [6–8]. This element plays a fundamental role in the photosynthetic electron transport chain (ETC) and therefore the conversion of light energy to organic carbon products [9–11].

Approaches currently used to measure phytoplankton primary production quantify rates at different points of the photosynthetic process (evolution of O_2_, assimilation of CO_2_, electron transport in photosystem II). These various rates can be decoupled in response to changes in environmental conditions or phytoplankton taxonomy [12]. For this reason, it is likely that iron limitation will affect the conversion factors between these various productivity metrics. Phytoplankton CO_2_-assimilation can be measured directly using the radioisotope tracer ^14^C [13,14]. This technique has been widely applied in biological oceanography over the past 60 years, despite a number of well-known limitations (e.g. low spatial and temporal resolution, high cost and labour intensity, bottle artifacts due to exclusion of grazers and contamination, requirement for radio-isotopes, ambiguity of whether net or gross production is measured [14–18]). In recent years, bio-optical approaches have emerged as an attractive alternative to overcome these limitations. Chlorophyll *a* fluorescence (ChlF) yields, measured by Pump and Probe, FRR, or PAM fluorometry, can be used to estimate rates of linear electron transport (i.e. rates of charge separation) in photosystem II (ETR_RCII_) [19–23], thus providing a measure of gross photosynthesis. Being non-intrusive, instantaneous and relatively inexpensive, these approaches can be used to examine phytoplankton photophysiology at unmatched spatial and temporal resolution, and improve the coverage of productivity estimates over vast oceanic domains.

Despite significant potential, active ChlF approaches are currently not widely applied to monitor rates of phytoplankton primary productivity. This is due, in part, to uncertainty in the conversion of ETR_RCII_ to ecologically relevant rates of CO_2_-assimilation [12, 24]. Numerous studies conducted over the past decades have collectively shown that the conversion factor linking ETR_RCII_ to CO_2_-assimilation in phytoplankton is not constant, but changes in response to taxonomy and environmental conditions [12, 20, 24–53]. On the physiological level, ETR_RCII_ and CO_2_-assimilation can be uncoupled by a number of energy-allocation processes that evolved to maximize photosynthetic efficiency while preventing photodamage. Marine phytoplankton evolved an exceptional photosynthetic plasticity to achieve this balance under low nutrient and fluctuating light conditions. A number of recent studies have examined this fine-tuning of electron transport and energy allocation within the phytoplankton photosynthetic apparatus, providing mechanistic insight into the processes decoupling CO_2_-assimilation and photosynthetic electron transport (e.g. [54–63]).

In this study, we examine the interacting effects of iron levels and instantaneous light availability on the coupling of ETR_RCII_ and CO_2_-assimilation in marine phytoplankton. We derived rates of ETR_RCII_ normalized to PSII reaction center content (mol e^-^ mol RCII^-1^ s^-1^), resulting in a conversion factor consisting of two parameters: the amount of chlorophyll *a* (chl *a*) functionally connected to each RCII (1/n_PSII_, mol chl*a* ^-1^ mol RCII^-1^), and the electron requirement for carbon fixation (Φ_e:C_, mol e^-^ mol C). Working with natural phytoplankton assemblages in the Northeast subarctic Pacific, and mono-specific phytoplankton cultures in the laboratory, we conducted simultaneous measurements of FRRF-derived ETR_RCII_ and ^14^C-based CO_2_-assimilation over a range of irradiances (P*vs*E curves) under high and low iron conditions. Our results demonstrate significant and interactive effects of irradiance and iron availability on the coupling of ETR_RCII_ and CO_2_-assimilation, with an increase in the conversion factor Φ_e:C_/n_PSII_ under excess light and low iron conditions. From a photophysiological point of view, increased decoupling appeared to be caused by the effects of increased excitation pressure on the photosynthetic ETC, resulting in a strong correlation between the derived conversion factor and the expression of non-photochemical quenching (NPQ) in the antennae of PSII. This correlation can, in turn, be used to derive rates of carbon-based productivity from FRRF data, without the need for any additional measurements.

## Methods

**Fig 1:**
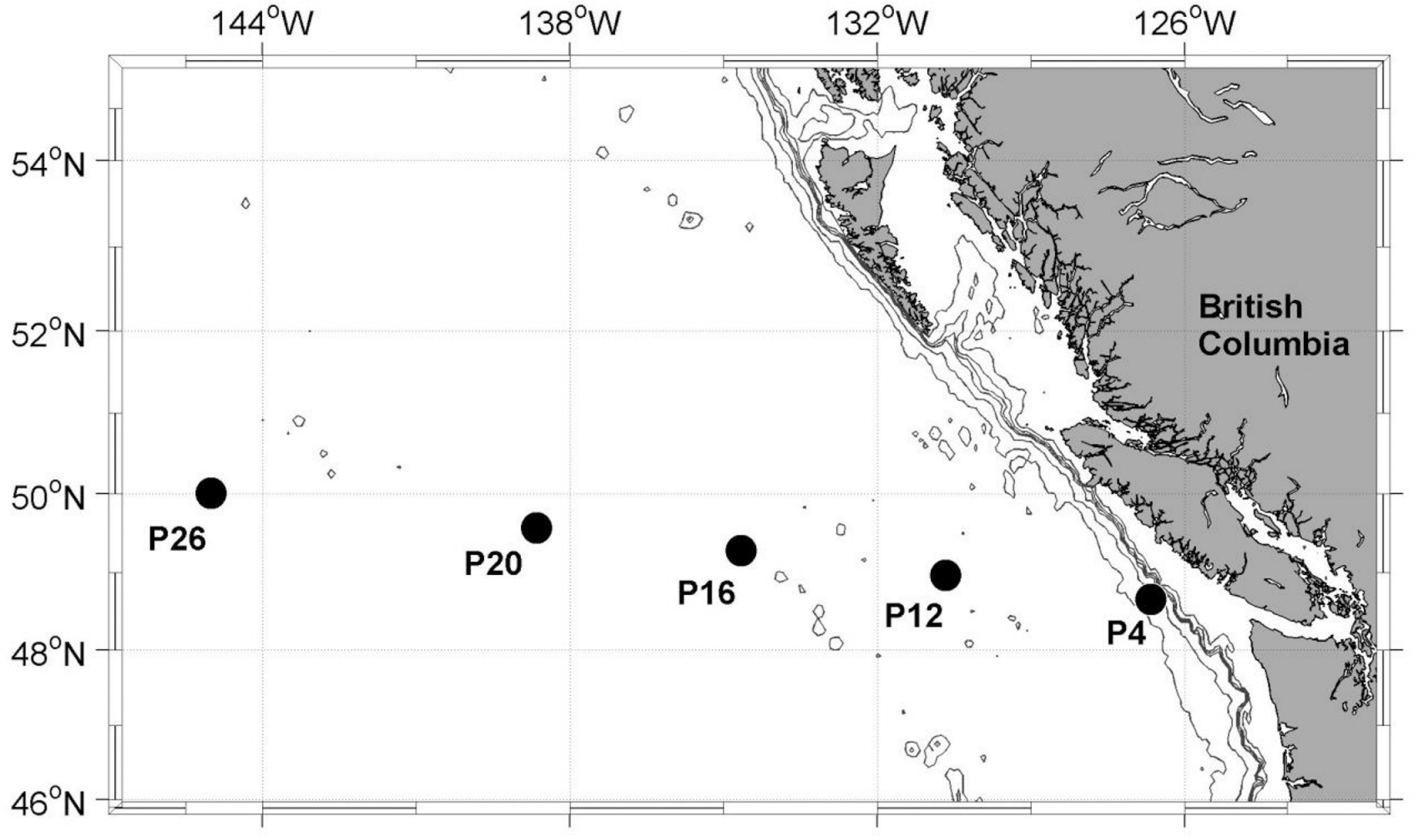
Map of sampling stations along the Line-P transect in the NE subarctic Pacific. The iron addition experiment was initiated at station P20, located in iron-limited high nutrient low chlorophyll (HNLC) waters. Sampling depths at other stations along the transect were: 30 m at P4; 5 m, 25 m and 40 m at P12, P16, P20 and P26.

In this study, we utilized three separate datasets. First, we examined the coupling of ETR_RCII_ and CO_2_-assimilation in a mixed phytoplankton assemblage during a 6 day ship-board iron addition experiment in iron-limited waters of the subarctic Pacific (Fig 1). Secondly, we conducted experiments with two mono-specific phytoplankton cultures grown under controlled light and iron conditions in the laboratory. These experiments were conducted to examine the physiological effects of iron and light on the conversion factor Φ_e:C_/n_PSII_, in the absence of potentially confounding taxonomic shifts. Finally, we applied the results obtained from the iron addition experiment to derive a conversion factor predicting rates of CO_2_-assimilation along a coastal to open ocean transect in the NE subarctic Pacific (Line-P, https://www.waterproperties.ca/linep/) (Fig 1). All fieldwork for this project was conducted under the authorization and permits of Fisheries and Oceans Canada.

### Iron addition experiment

All fieldwork was conducted on board the *CCGS John P*. *Tully* in August—September 2013. A 6 day iron addition experiment was initiated at P20 (49°34 N, 138°40 W) (Fig 1), located in iron-limited HNLC waters. Water was collected before dawn from 7 m depth using a trace metal clean pumping system and an on-deck class 100 laminar flow hood (cf. [64]). In order to eliminate macro-zooplankton, the water was pre-filtered through acid washed 200 μm Nitex mesh. Six trace metal-cleaned 10 L cubitainers were rinsed and filled in random order. Triplicate iron-addition treatments were amended with 1 nM Fe (ammonium iron (II) sulfate hexahydrate ((NH_4_)_2_Fe(SO_4_)_2_·6H_2_O), dissolved in 0.05M HCl), while triplicate controls were left unamended. Cubitainers were kept in on-deck incubators continuously supplied with seawater pumped from 5 m depth. Light intensity was adjusted to ~ 50% of full sunlight with neutral density screening and irradiance was continuously logged using a LI-1000 radiation sensor (LI-COR, USA), located 2 m above the incubator. This level of light reduction was chosen to avoid exposing the phytoplankton to irradiances higher than in situ values. On days 1, 3 and 5 at exactly 2 hours after local sunrise, 500 mL of water were sub-sampled from each cubitainer using trace metal clean techniques. Sub-samples were analyzed for total chlorophyll *a* concentration ([chl *a*]), photophysiological parameters and rate measurements as outlined below. On the last day of the experiment, additional samples were collected for pigment analysis by high pressure liquid chromatography (HPLC), and the determination of absorption spectra using the quantitative filter technique (QFT) [65].

### Laboratory culturing

The oceanic centric diatom *Thalassiosira oceanica* (CCMP isolate 1003, Sargasso Sea) and the oceanic prymnesiophyte *Chrysochromulina polylepis* (NEPCC isolate 242, NE subarctic Pacific) were grown under iron-replete and iron-limiting conditions. We chose these two species as representative eukaryotic open ocean species, common in the region of our field study [66,67]. Iron-limited growth conditions were chosen to achieve an approx. 50% reduction in growth rate. Both species were cultured in 28 mL acid-cleaned polycarbonate tubes using the artificial seawater medium AQUIL [68], prepared as described by Maldonado et al. [69]. All cultures were kept at 19°C in continuous, sub-saturating light (ca. 40 μmol quanta m^-2^ s^-1^). Growth was monitored by daily measurements of in vivo chl *a* fluorescence using a Turner 10-AU Fluorometer, and cultures were kept in exponential growth phase using semi-continuous batch culturing [70]. Cultures were considered acclimated when growth rates during ca. 40 cell divisions (five successive transfers), varied by <15% [70]. Acclimated, exponentially growing cells were used to inoculate triplicate 200 mL cultures. These 200 mL cultures were sub-sampled several times for FRRF measurements (see below), which demonstrated that cells maintained steady-state photophysiology throughout the sampling phase. During early to mid-exponential phase, each replicate culture was sampled for duplicate ETR_RCII_-P*vs*E curves, duplicate ^14^C-P*vs*E curves and triplicate [chl *a*] samples. Sterile, trace metal clean techniques were used at all times.

### Station sampling

In addition to the iron addition experiment, seawater samples were collected at five hydrographic stations (P4, P12, P16, P20, and P26) spanning a coastal to open ocean transect in the NE subarctic Pacific (Fig 1). Collection of water column hydrographic profiles was performed with a CTD (SeaBird Electronics, model 911 plus) equipped with a dissolved oxygen sensor (SBE 43), fluorometer (Seapoint), and an underwater photosynthetically active radiation (PAR) sensor (Biospherical QSP-400). At each of the stations, water was collected from Niskin bottles at three depths exactly two hours after local sunrise and processed immediately for rate measurements, photophysiological parameters, and [chl *a*] as described below.

### [chl *a*]

For the 3 sets of experiments outlined above, samples for [chl *a*] were filtered onto pre-combusted 25 mm glass fiber filters (GF/F) using low vacuum pressure (<5 mm Hg) and analyzed following the method of Welschmeyer [71]. In the field, triplicate 100–300 mL samples were filtered and stored at -20°C until analysis within three weeks of collection. In the laboratory, triplicate culture samples (10 mL, 20 mL and 30 mL) were collected and analyzed immediately. Each sample was analyzed in duplicate.

### Carbon assimilation

For both laboratory and field work, rates of carbon assimilation were measured as small volume P*vs*E curves in a custom built photosynthetron [72]. In the field, 300 mL of water were spiked with 150 μCi NaH^14^CO_3_ (final concentration 0.5 μCi mL^-1^, 52.5 mCi mL^-1^ specific activity) (Perkin-Elmer) immediately after sampling. Spiked samples were mixed gently but thoroughly, aliquoted into 20 ml glass scintillation vials and placed into the photosynthetron. Temperature was kept within 1°C of in situ temperature by circulating water from a water-bath through an aluminum cooling jacket (the offset from in situ temperature was larger for station samples because samples from different depth had to be incubated simultaneously). Light was provided by high power light emitting diodes (LEDs) located under each scintillation vial. Each P*vs*E curve consisted of 11 light levels spanning intensities from 3 to 600 μmol quanta m^-2^ s^-1^. Actual light intensities were measured before and after each experiment using a 4π quantum sensor (QSL-2100, Biospherical Instruments) immersed in water inside a scintillation vial. Incubations lasted for 3–4 hours and were ended by gentle filtration onto 25 mm GF/F filters. Filters were pre-combusted to reduce nominal pore size to approx. 0.4 μm. For each curve, three time-zero samples were taken by filtering 20 mL immediately after spiking. The total ^14^C activity added was determined from three 1 mL aliquots of the spiked sample added to 1 mL 1 M NaOH. All work was done under low light and filters were stored in scintillation vials at -20°C until processing within 1 month of the experiment. During laboratory processing, 500 μL of 3 M HCl was added to each filter and vials were left to degas for >24 hours to eliminate any inorganic ^14^C remaining in the samples. Ten mL of scintillation cocktail (Scintisafe plus, Fisher) were added to each vial, and vials were then vortexed and left to stand in the dark for >12 hours before analysis on a liquid scintillation counter (Beckman). Disintegrations per minute (DPM) were derived from scintillation counts using a quench curve prepared from commercial ^14^C standards (Perkin-Elmer). DPM were converted to units of carbon biomass following Knap et al. [73].

The ^14^C protocol used for laboratory cultures was the same as outlined above with the following exceptions. We spiked 80 mL of exponentially growing culture with 40 μCi NaH^14^CO_3_ and 3 mL aliquots were incubated in the photosynthetron for 30 minutes. Duplicate curves were measured for each sample. The incubation was terminated by adding 1 mL of 1 M HCl to each vial and samples were dried completely, omitting the filtration step. After drying, salts were re-suspended in 1 mL MilliQ water. For both laboratory and field measurements ^14^C-P*vs*E curves were fit following Webb et al. [74], as described below.

### Chl *a* fluorescence parameters and ETR_RCII_

A bench-top FRRF instrument (Soliense Inc.) was used for all active ChlF measurements. In the field, opaque bottles were used for sub-sampling from the rosette or iron addition experiment, and light in the laboratory was kept low at all times to allow oxidation of the ETC and relaxation of NPQ. For all measurements, background fluorescence blanks were prepared by very gently filtering a small amount of sample through a pre-combusted GF/F. Single turnover (ST) flash protocols consisted of 100 flashlets with 1.0 μs length and 2.5 μs interval (46200 μmol quanta m^-2^ s^-1^ peak power intensity, resulting in a ST flash length of 250 μs, providing ~5–10 quanta per RCII). The excitation power was selected at the beginning of the cruise to saturate the observed fluorescence transients within the first half of the ST excitation protocol. Our experience indicates that this approach offers the best signal-to-noise ratio in the recovered parameters, while accommodating significant variations in the photosynthetic properties of the local phytoplankton populations along the cruise track, without re-adjusting of the excitation protocol. Excitation power was provided by an array of eight LEDs at four wavelengths centered on 445 nm, 470 nm, 505 nm, and 530 nm (equal intensity at each wavelength; see S1 Fig for more information on the spectral distribution). We measured steady state light curves (SSLC), where each sample was exposed to 10 actinic ‘background’ irradiances ranging from 0 to 1000 μmol quanta m^-2^ s^-1^, also provided at four wavelengths (S1 Fig). The relatively long duration of the SSLCs in this study could create some potential for the settling of cells which could influence the ChlF yield. However, our sampling region is known to be dominated by small cells [66], which should have a slow settling rate. Equally, the laboratory isolates used during this study stay in suspension for many hours, and it is thus unlikely that rapid settling of cells drastically altered our results.

All ChlF yields and parameters described below were derived by an iterative non-linear fitting procedure, applying the four parameter biophysical model of Kolber et al. [21] to a mean of 20 consecutive ST flashes using custom software (Z. Kolber). This software accounts for a formation of fluorescence quenching, most likely due to formation of a P680 triplet, which reduces the maximum fluorescence yield attainable during the ST flash by 3–6%. Throughout the SSLC, ST flashes were applied continuously (at 1 s interval), while the length of each light step was optimized to allow all derived parameters to reach steady state (ca. 5 min). ChlF yields and parameters corresponding to each light level were obtained from the mean of the last three acquisitions at each light level. In this way, we derived the fluorescence yields F_o_ and F_m_ (in dark regulated state) as well as F′ and F_m_′ (in the light regulated state for each light level of the SSLC). F_o_′ was calculated as F_o_′ = F_o_/(F_v_/F_m_ + F_o_/F_m_′) [75]. Even though this derivation has become widely accepted in the literature, we caution here that it might not hold for values derived under high background irradiance (see [76]) and varying stress levels experienced by natural phytoplankton assemblages.

Five fluorescence signals, F_o_, F_m_, F′, F_m_′ and F_o_′ were used to calculate ChlF parameters, following Roháček [77]. In the dark-regulated state, we derived the commonly used F_v_/F_m_ ratio as F_v_/F_m_ = (F_m_-F_o_)/F_m_ [78]. For each light level of the SSLC protocol we have calculated the following ChlF parameters: (1) The photochemical quenching of variable fluorescence, F_q_′/F_v_′ = (F_m_′-F′)/(F_m_′-F_o_′), which quantifies the fraction of functional RCII in the open state (i.e. primary quinone acceptor Q_A_ in the oxidized state) [79]. (2) The maximum quantum yield of PSII photochemistry, F_v_′/F_m_′ = (F_m_′-F_o_′)/F_m_′, which can be used to quantify the extent to which photochemistry in PSII is limited by competition with thermal decay of excitation energy [75]. (3) The overall quantum efficiency of photochemical energy conversion in PSII at a given light intensity (note that numerous definitions for this parameter exist in the literature), F_q_′/F_m_′ = (F_m_′-F′)/F_m_′ = Ф_PSII_′ (the product of F_q_′/F_v_′ and F_v_′/F_m_′ [19]). Furthermore, the functional absorption cross section of PSII, σ_PSII_ (Å^2^ RCII^-1^), was derived from the rate of closure of RCII in the dark-regulated and at each light-regulated state [20,21]. The connectivity parameter, ρ, was also calculated, but not used in our analysis.

Rates of charge separation (i.e. ETR_RCII_) in functional RCII (mol e^-^ mol RCII^-1^ s^-1^) were estimated as the product of incident irradiance (E), the fraction of irradiance absorbed by PSII (σ_PSII_) and the efficiency with which charge separation occurs in RCII. We calculated ETR_RCII_ as

ETRRCII=E*σPSII′*Fq′Fv′*6.022*10−3 (1)

where E (μmol quanta m^-2^ s^-1^) is the actinic irradiance at each light level, σ_PSII_′ (Å^2^ RCII^-1^) is the functional absorption cross section at E and F_q_′/F_v_′ is the photochemical capacity of PSII at E. The number 6.022 x 10^−3^ converts μmol quanta to quanta and Å^2^ to m^2^. Because of potential systematic errors in the calculation of F_o_′, we also calculated ETR_RCII_ as

$${\text{ETR}}_{\text{RCII}}={\text{E}\;*\;\sigma }_{\text{PSII}}\;*\;\frac{\left({\text{F}}_{\text{q}}\prime /{\text{F}}_{\text{m}}\prime \right)}{\left({\text{F}}_{\text{v}}/{\text{F}}_{\text{m}}\right)}\;*\;6.022\;*\;10^{-3}$$ (2)

which does not require the knowledge of F_o_′. Both calculations are equivalent, assuming that non-photochemical quenching processes affecting ChlF can be adequately accounted for in either the absorption term (Eq 1) and the efficiency term (Eq 2). While Eq 2 does not require F_o_′ (which was not measured directly) or σ_PSII_′ (which is difficult to derive at high irradiances), it does rely on parameters measured in the fully dark-regulated state, which can be difficult to achieve in field assemblages. For all ETR_RCII_ calculated during our iron addition experiment (n = 345) the difference between values calculated in both ways ranged from 0.5 to 21% with a mean coefficient of variance of 5.5%. Both approaches thus provided similar results in the analysis of our data, and the differences observed were not systematically related to the treatment (high *vs* low Fe).

Non-photochemical quenching (NPQ) at each light level was estimated as the normalized Stern-Volmer quenching coefficient, defined as NPQ_NSV_ = (F_m_′/F_v_′)-1 = F_o_′/F_v_′ [65]. Quantification of NPQ using NPQ_NSV_ instead of the more commonly used Stern-Volmer coefficient of quenching, defined as NPQ_SV_ = (F_m_-F_m_′)/F_m_ [80], is appropriate for our data-set, as it resolves differences between NPQ present in the dark-regulated state.

### P*vs*E curves

Measurements of CO_2_-assimilation and ETR_RCII_ were plotted against irradiance, and the exponential model of Webb et al. [74] was fit to the data using a non-linear least squares regression procedure in Matlab. For the CO_2_-assimilation data, an intercept parameter was added to force the regression through the origin and provide a good fit in the linear part of the P*vs*E curve [28,81]. For both rates of productivity, we derived the light saturated maximum rate P_max_ and the light utilization efficiency α. When photoinhibition was observed at high irradiances, the data-points were excluded from the fitting procedure.

### Derivation of conversion factor

Because we derived ETR_RCII_ in units of mol e^-^ mol RCII^-1^ s^-1^ and CO_2_-assimilation in units of mol C mol chl *a* ^-1^ s^-1^, the conversion factor between the two rates accounts for changes in chl *a* functionally associated with each RCII (1/n_PSII_, mol chl *a* mol RCII^-1^) and the number of charge separations in RCII needed per CO_2_-assimilated into organic carbon products (Φ_e:C_, mol e^-^ mol C^-1^).

ETRRCII(mol e−mol RCII−1s−1)CO2assimilation(mol C mol chla−1s−1)=Φe:C(mol e−mol C)*1nPSII(mol chlamol RCII) (3)

In this approach, we attribute the observed decoupling between ETR_RCII_ and CO_2_-assimilation to changes in both, 1/n_PSII_ and Φ_e:C_. We recognize that combining Φ_e:C_ and 1/n_PSII_ into one conversion factor obscures the mechanistic underlying of the observed decoupling. Nevertheless, as we will show, our approach has the potential to provide FRRF-derived estimates of phytoplankton primary productivity in carbon units without the need for many of the auxiliary measurements and inherent assumptions used in previous studies.

The value of 1/n_PSII_ is known to change significantly as a function of taxonomy [22], light [22,82], macro-nutrients [83], and iron availability [84–90]. Therefore we could not assume a constant value for 1/n_PSII_, as has been done in most previous studies [24]. Although 1/n_PSII_ can be directly measured from oxygen flash yield experiments (e.g. [91–93]), the approach is labour-intensive and not practical for routine field sampling. A new approach to derive [RCII] directly from FRRF measurements has been developed [94,95], but not implemented in our study because the inherent assumption that the ratio of rate constants of photochemistry and fluorescence (k_p_/k_f_) is confined to a narrow range, does not hold under varying levels of iron limitation [62,87,94].

Having established a relationship between light intensity and rates of CO_2_-assimilation and ETR_RCII_ for each sample, we were able to model the light dependency of the conversion factor Φ_e:C_/n_PSII_. This approach allowed us to observe how the coupling of ETR_RCII_ and CO_2_-assimilation is modulated by incident irradiance, and how, in turn, iron limitation influences the light-dependent response. Additionally, we used α and P_max_ of each rate to derive the conversion factor under sub-saturating and saturating light conditions, respectively.

## Results

### Effect of iron addition on phytoplankton community composition, photophysiology, ETR_RCII_ and CO_2_-assimilation in the NE subarctic Pacific

**Fig 2:**
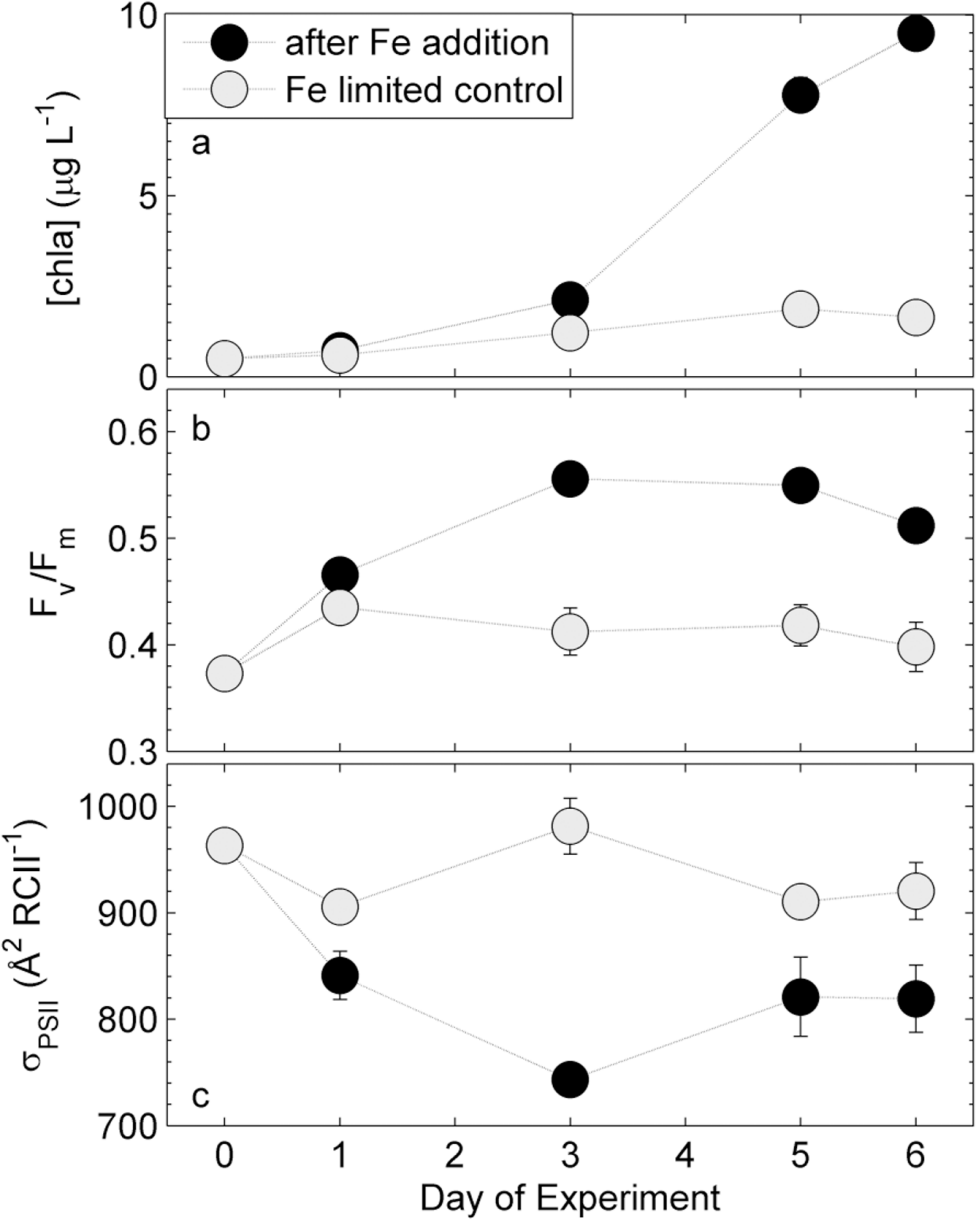
Response of chl *a* biomass and photophysiology during the on-board iron addition experiment. Shown are changes in (a) [chl *a*], (b) F_v_/F_m_, and (c) σ_PSII_. Error bars represent standard errors from three biological replicates and are sometimes smaller than the symbol.

Phytoplankton assemblages at station P20 in the NE subarctic Pacific (Fig 1) responded strongly to iron addition in a ship-board incubation experiment (Fig 2). Six days after iron addition, [chl *a*] increased by an order of magnitude, whereas the control (i.e. no iron addition) showed only a small increase in [chl *a*]. This result confirms that the initial phytoplankton assemblage was iron-limited (Fig 2A), and that we were able to carry out the manipulation experiment without significant contamination of the control bottles. The slight increase in [chl *a*] in the control treatments is likely attributable to a decrease in grazing pressure and to changes in the light environment (i.e. lower and less fluctuating light). Iron addition also significantly affected phytoplankton photophysiology, as demonstrated by rapid changes in the parameters σ_PSII_ and F_v_/F_m_ derived in the dark-regulated state (Fig 2B and 2C). F_v_/F_m_ initially increased in both treatments, but then remained low in the control while continuing to increase in the iron addition treatment (Fig 2B). While the functional absorption cross-section of PSII, σ_PSII_ (Å^2^ RCII^-1^), remained high and relatively constant in the iron-limited control, it declined rapidly after iron addition, and remained ~25% lower than that of the initial phytoplankton assemblage (Fig 2C). The observed changes in F_v_/F_m_ and σ_PSII_ may have resulted from both, photophysiological responses and from changes in species composition. CHEMTAX analysis of pigments sampled on day 6 of the experiment showed that the addition of iron changed the taxonomic composition of the phytoplankton assemblage (S2 Fig). Most prominently, the abundance of chlorophytes decreased from 7% to 1%, prymnesiophytes decreased from 55% to 22%, pelagophytes increased from 17% to 39%, and diatoms increased from 1% to 16% in iron amended bottles. A similar response has been observed in previous iron addition experiments conducted in this region [96].

**Fig 3:**
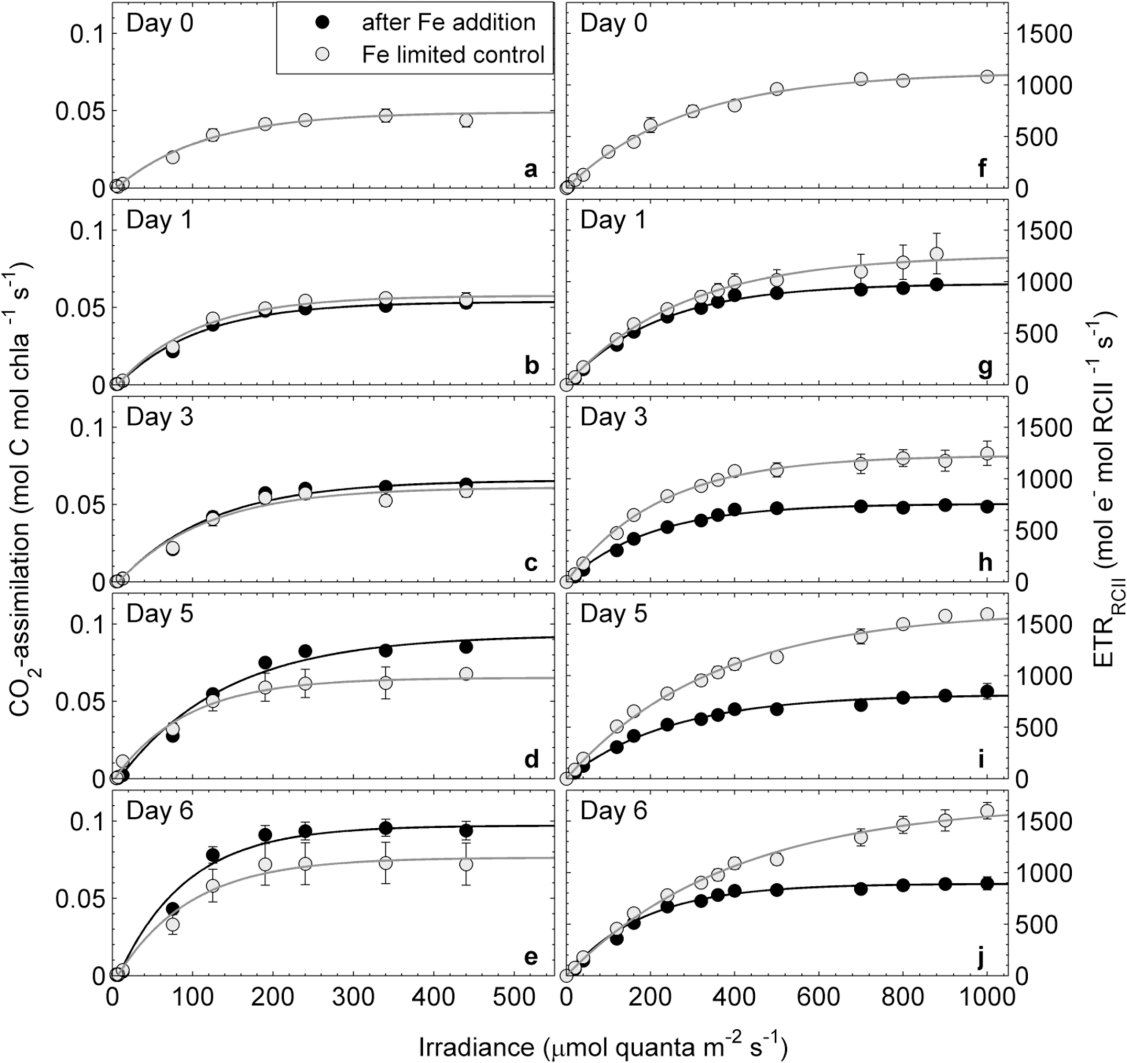
Response of rates of CO_2_-assimilation (mol C mol chl *a* ^-1^ s^-1^) and ETR_RCII_ (mol e^-^ mol RCII ^-1^ s^-1^) during the iron addition experiment. Both rates were measured as a function of irradiance, and P*vs*E curves were fit with the exponential model of Webb et al. [74]. Shown are mean values from three biological replicates where error bars represent standard error of mean and are sometimes smaller than symbols.

We measured P*vs*E curves of short-term CO_2_-assimilation and ETR_RCII_ five times during the iron addition experiment (Fig 3). Both rates show the expected light dependency, and were affected by iron addition. However, the response to iron addition differed for CO_2_-assimilation and ETR_RCII_. Chlorophyll *a*-normalized CO_2_-assimilation showed a small, though not statistically significant, increase after iron addition (Fig 3A–3E). The observed increase in the chl *a*-normalized rate was small, because cellular chl *a* content increased in parallel with CO_2_-assimilation (under all nutrient limitations, cellular chl *a* in phytoplankton is drastically reduced, a condition referred to as chlorosis, e.g. [97]). The strong effect of iron addition on CO_2_-assimilation can be seen more clearly when rates are normalized to volume. Indeed, volume-normalized CO_2_-assimilation rates increased more than 8-fold after iron addition in this experiment (S3 Fig). In contrast to rates of CO_2_-assimilation, ETR_RCII_ decreased significantly after iron addition, when compared to the iron-limited control treatment (Fig 3F–3J).

**Fig 4:**
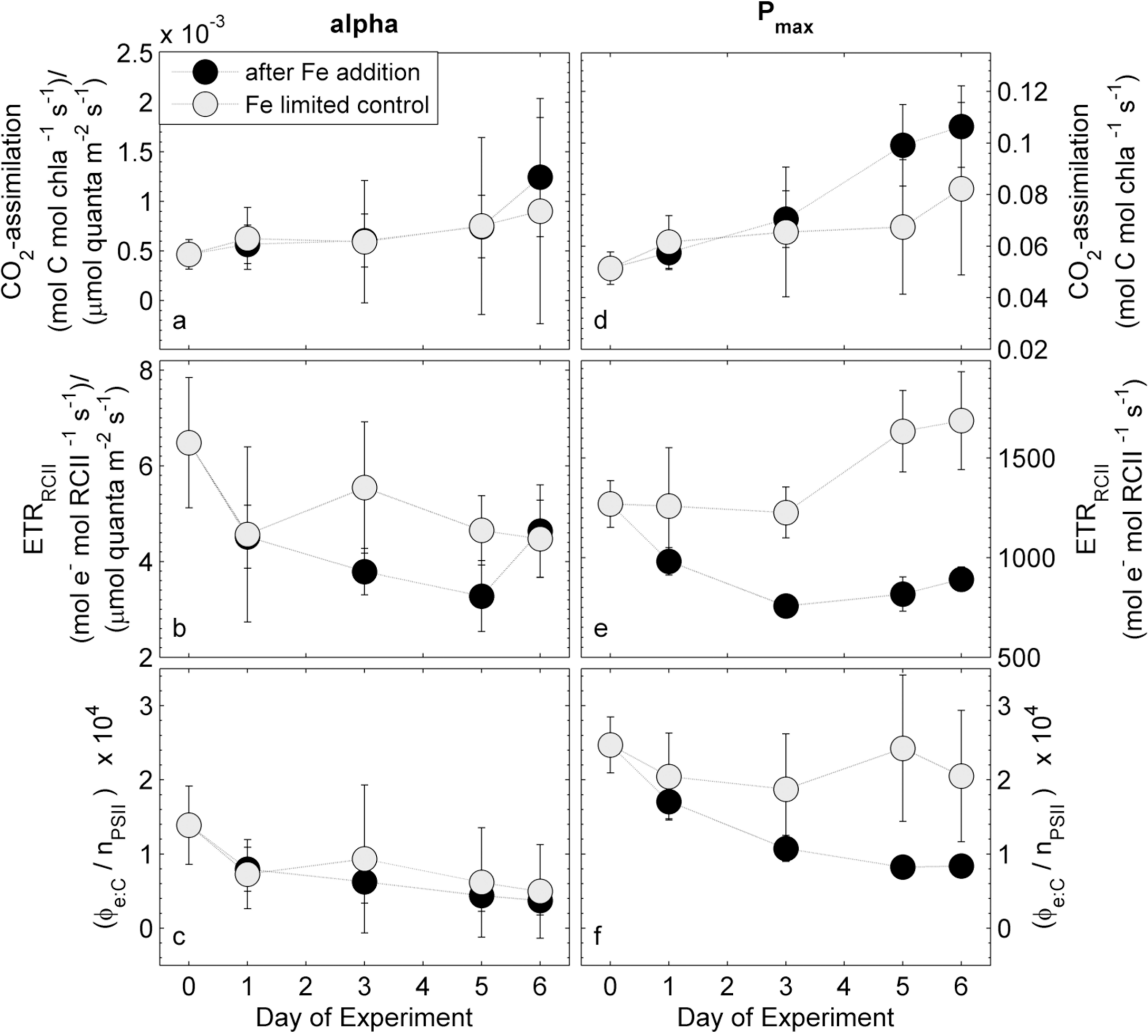
Time-course of α (a-c) and P_max_ (d-f) of CO_2_-assimilation, ETR_RCII_ and the derived conversion factor Φ_e:C_/n_PSII_ during the iron addition experiment. The conversion factor Φ_e:C_/n_PSII_ under light limiting conditions is derived from values in (a) and (b). Similarly, the conversion factor Φ_e:C_/n_PSII_ at light saturation is derived from the values in (d) and (e). The error in (a), (b), (c), and (d) is the 95% confidence interval of the parameter derived from the fit to data from three biological replicates, and the error in (c) and (f) is the propagated error from (a)/(b) and (d)/(e), respectively.

The response of CO_2_-assimilation and ETR_RCII_ to iron addition is further visualized in Fig 4, which shows changes in light-limited slopes (α) and light saturated rates (P_max_), as well as the derived conversion factor Φ_e:C_/n_PSII_ for α and P_max_, throughout the experiment. Values for α and P_max_ were derived from the ^14^C-based and FRRF-based P*vs*E curves shown in Fig 3. No statistically significant change in values of α could be determined for either chl *a*-normalized CO_2_-assimilation, ETR_RCII_ or Φ_e:C_/n_PSII_ (p-value > 0.05). Similarly, the P_max_ for chl *a*-normalized CO_2_-assimilation remained relatively constant in the control, and did not show a statistically significant increase after iron addition (p-value > 0.05) (Fig 4D). In contrast, there was a significant (p-value < 0.05) decrease in P_max_ for ETR_RCII_ following iron-addition, as compared to the control treatments, which exhibited a small increase in this variable over the course of the experiment (Fig 4E). The observed changes in the P_max_ for CO_2_-assimilation and ETR_RCII_, resulted in a decrease in Φ_e:C_/n_PSII_ in the iron addition treatment compared to the relatively constant value observed in the iron-limited control (Fig 4F). This difference was statistically significant for the last 2 days of the experiment (p-value < 0.05). When compared to the initial value on day 0 of the incubation, the conversion factor Φ_e:C_/n_PSII_ for P_max_ decreased by 66% after iron addition, and by 16% in the iron-limited control (Fig 4F). These results indicate that the iron-dependent changes in Φ_e:C_/n_PSII_ are most readily apparent under high irradiance conditions where photosynthesis is light-saturated.

**Fig 5:**
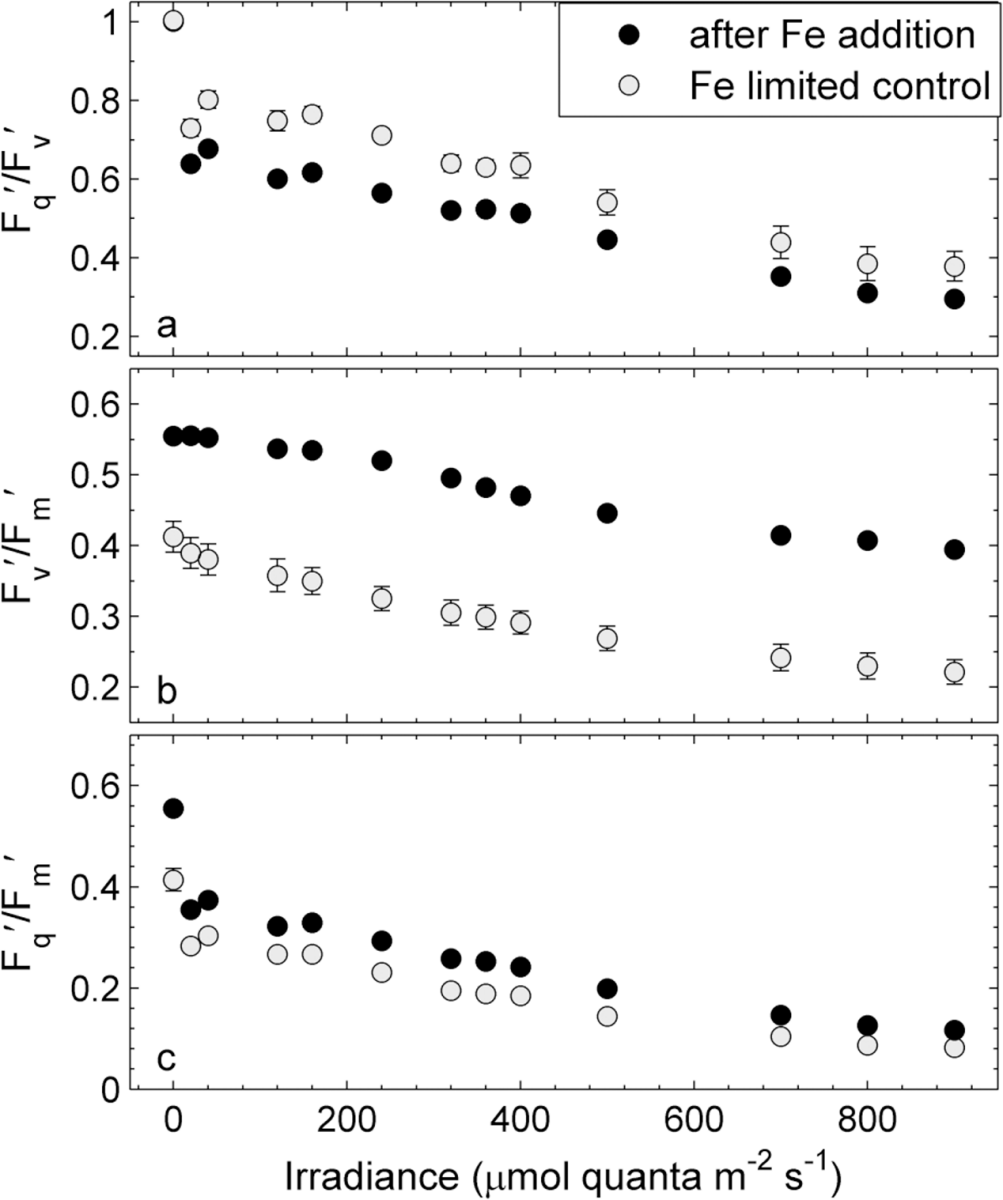
Light dependency of ChlF-derived parameters from FRRF measurements on day three after iron addition and in the iron-limited control treatment. The parameter F_q_′/F_v_′ (a) represents the efficiency of charge separation in functional RCII and is an estimate of the fraction of open RCII (i.e. Q_A_ oxidized) at any given light level. The parameter F_v_′/F_m_′ (b) represents the efficiency of excitation energy capture by the fraction of open RCII and can be used to quantify the extent to which non-photochemical quenching in the PSII antenna competes with photochemistry for excitation energy. The parameter F_q_′/F_m_′ (c) represents the overall quantum efficiency of photochemical energy conversion in PSII (Φ′_PSII_). See text for a full description of these parameters and their interpretation. Error bars represent standard errors from three biological replicates and are often smaller than symbols.

To better explain the iron-dependent decrease in ETR_RCII_ and Φ_e:C_/n_PSII_ observed in our data, we examined changes in additional FRRF-derived ChlF parameters, measured on day 3 after iron addition. We choose day 3 for the in-depth analysis of our data, but trends observed on this day were representative of those observed throughout the experiment. The parameter F_q_′/F_v_′ represents the efficiency of charge separation in functional RCII (Fig 5A). It is an estimate of the fraction of open RCII (i.e. Q_A_ oxidized) at any given light level, and therefore always equals one at zero irradiance. On day 3 after iron addition, we observed higher F_q_′/F_v_′ for the iron-limited control at all irradiance levels (Fig 5A), indicating a greater fraction of open reaction centers. The parameter F_v_′/F_m_′, the efficiency of excitation energy capture by the fraction of open RCIIs [19], can be used to quantify the extent to which photochemistry in RCII is limited by thermal energy dissipation in the antenna [75]. This parameter was significantly reduced in the iron-limited control relative to the iron addition treatment (Fig 5B), indicating that the efficiency of excitation energy transfer in the light-harvesting antenna was comprised. The overall efficiency of charge separation per quantum absorbed in PSII (F_q_′/F_m_′) is the product of F_q_′/F_v_′ and F_v_′/F_m_′ [19,77]. On day 3, at all light levels, F_q_′/F_m_′ was higher in the iron addition treatment than in the iron-limited control (Fig 5C).

**Fig 6:**
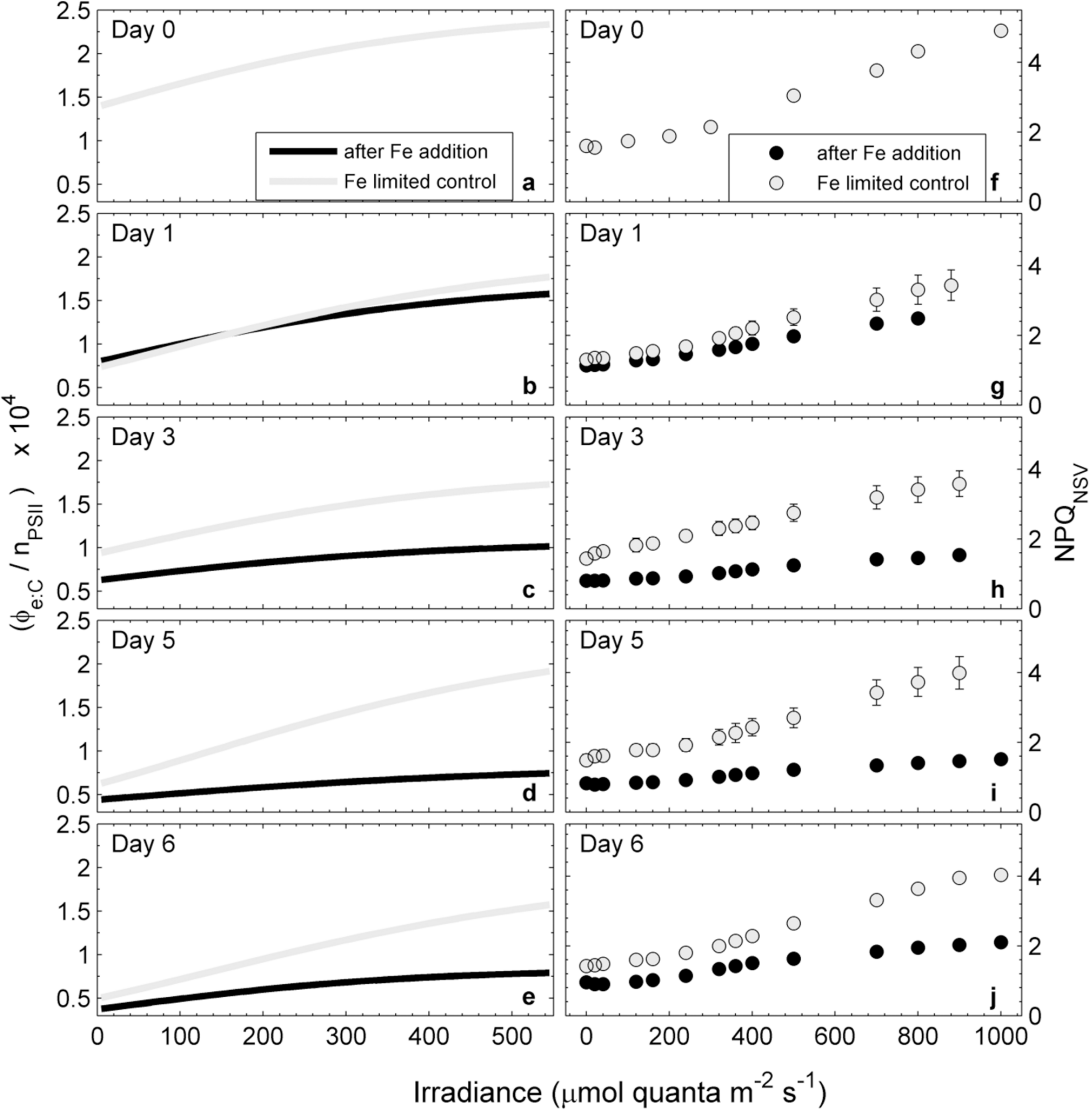
Changes in the light dependency of the conversion factor Φ_e:C_/n_PSII_ (a-e) and NPQ_NSV_ (f-j) over the course of the iron addition experiment. Units of in Φ_e:C_/n_PSII_ are (mol e- mol C) / (mol chl *a* mol RCII^**-1**^). The curves were derived by dividing corresponding values of ETR_RCII_ and CO_2_-assimilation from the P*vs*E curves presented in Fig 3. NPQ was estimated as the normalized Stern-Volmer quenching coefficient NPQ_NSV_ = F_o_′/F_v_′ and is unitless [65]. Error bars are the standard error from three biological replicates and often smaller than symbols.

We used our P*vs*E measurements of CO_2_-assimilation and ETR_RCII_ to examine the light-dependent response of the conversion factor Φ_e:C_/n_PSII_. Our results (Fig 6) show that Φ_e:C_/n_PSII_ increased with increasing irradiance, regardless of iron treatment and day of the experiment (Fig 6A–6E). However, this light-dependent increase was much more pronounced in the iron-limited control treatment. It is important to note that the magnitude and light-dependency of Φ_e:C_/n_PSII_ in the iron-limited control treatment changed over the course of the experiment relative to the initial sample (Fig 6A). This shift in Φ_e:C_/n_PSII_ in the absence of iron addition likely reflects changes in light quality and quantity in the incubation bottles relative to the ambient water column.

Also shown in Fig 6 is the light and iron dependency of NPQ_NSV_, estimated as F_o_′/F_v_′. This parameter showed a light and iron-dependent response that was remarkably similar to Φ_e:C_/n_PSII_, with values increasing with increasing light, regardless of treatment and day of the experiment, and decreasing in response to iron addition (Fig 6F–6J). The NPQ_NSV_ values measured in our initial sample (Fig 6F) were higher than those measured in either control or iron addition treatments during the following days. We attribute this effect to a more stable light environment in the incubation bottles, relative to in situ irradiance levels.

**Fig 7:**
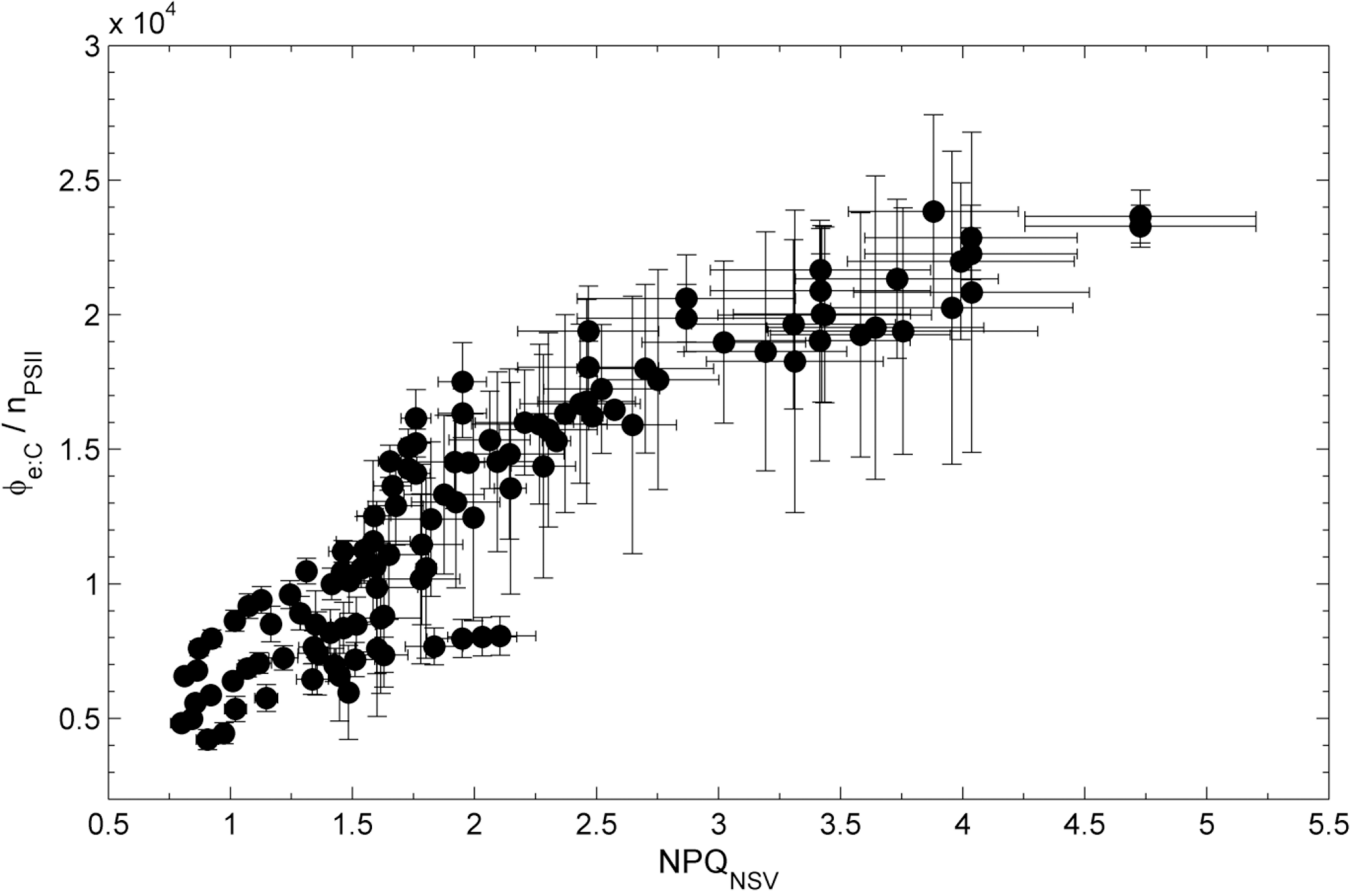
Relationship between the conversion factor Φ_e:C_/n_PSII_ and NPQ_NSV_ values during the iron addition experiment. Values of Φ_e:C_/n_PSII_ were derived from P*vs*E curves of CO_2_-assimilation and ETR_RCII_ at irradiances corresponding to each ETR_RCII_-P*vs*E curve light level. Units of Φ_e:C_/n_PSII_ are (mol e^**-**^ mol C^**-1**^) / (mol chl *a* mol RCII^**-1**^). NPQ_NSV_ values were derived as F_o_′/F_v_′ for each light level of the SSLC. Data points represent means and standard errors for parameters derived from three biological replicates. A quadratic fit through all data points (Φ_e:C_/n_PSII_ = -733.21 NPQ^**2**^+8792.4 NPQ– 1477.1) is statistically significant (R^**2**^ = 0.70, p-value < 0.0001).

Given the similar light and iron-dependent responses of Φ_e:C_/n_PSII_ and NPQ_NSV_, we sought to examine the relationship between these two variables. In order to do so, however, it was necessary to derive NPQ_NSV_ and Φ_e:C_/n_PSII_ values at a standard set of light levels, matching those of the FRRF derived ETR_RCII_-P*vs*E curves. For each sample, ETR_RCII_-P*vs*E curves consisted of 14 light levels spanning from 0 to 1000 µmol quanta m^-2^ s^-1^. These light levels did not exactly match those used for the CO_2_-assimilation experiments. We thus used the P*vs*E curve fits of our ^14^C data to derive the CO_2_-assimilation values at light levels matching those of the ETR_RCII_-P*vs*E curves. In this way, we were able to compile a dataset of 298 paired values for NPQ_NSV_ and Φ_e:C_/n_PSII_, derived from 27 sets of ETR_RCII_ and ^14^C P*vs*E curves during the iron addition experiment. Plotting these Φ_e:C_/n_PSII_ values against the corresponding NPQ_NSV_ reveals a strong and statistically significant correlation (R^2^ = 0.70, p-value < 0.0001, for quadratic fit) (Fig 7).

### Effects of iron limitation on photophysiology and rates of ETR_RCII_ and CO_2_-assimilation in mono-specific phytoplankton cultures

Using methods analogous to those applied to mixed phytoplankton assemblages in the NE subarctic Pacific; we measured P*vs*E curves of CO_2_-assimilation and ETR_RCII_ in mono-specific laboratory cultures of two open ocean phytoplankton species. The results, summarized in Table 1, show similar trends as observed in our field data. Steady-state growth rates (μ, d^-1^) in the low iron cultures were 68% and 49% of iron-replete growth rates in *T*. *oceanica* and *C*. *polylepis*, respectively (Table 1). For both species, F_v_/F_m_ in iron-limited cultures was reduced (by 32% and 20% in *T*. *oceanica* and *C*. *polylepis*, respectively). In iron-limited *T*. *oceanica*, σ_PSII_ increased by 15%, while it increased by 5% in *C*. *polylepis*. The iron dependent changes in μ, F_v_/F_m_ and σ_PSII_ was statistically significant in both species (one tailed p-value < 0.0001 and < 0.01 for *T*. *oceanica* and *C*. *polylepis*, respectively). Chlorophyll *a*-normalized CO_2_-assimilation at P_max_ remained relatively constant in both species (p-value > 0.05). In contrast, we observed a 90% increase in ETR_RCII_ at P_max_ in *T*. *oceanica* under iron-limited growth conditions. *C*. *polylepis* also exhibited an increase in ETR_RCII_ at P_max_ under iron-limited conditions, but this increase was not statistically significant (p-value > 0.05). Regardless of species-specific differences, both species showed the same trend of increased Φ_e:C_/n_PSII_ and NPQ_NSV_ under iron limitation (Table 1), which is consistent with our field observations. Furthermore, the species-specific differences observed in our laboratory experiments are consistent with changes in phytoplankton assemblage composition observed in our iron addition experiment, where the abundance of diatoms (lower Φ_e:C_/n_PSII_) was increased in the iron addition treatment and the abundance of prymnesiophytes (higher Φ_e:C_/n_PSII_) was decreased (S2 Fig).

**Table 1:** Effect of iron limitation on photophysiology in two mono-specific phytoplankton cultures grown in the laboratory.

	*T*. *oceanica*		*C*. *polylepis*	
[Fe]	42nM	0.13nM	42nM	1.28nM
μ (d^-1^)	1.27 ± 0.14 (n = 6)***	0.41 ± 0.09 (n = 5)	0.53 ± 0.12 (n = 5)**	0.27 ± 0.05 (n = 4)
F_v_/F_m_	0.63 ± 0.01***	0.43 ± 0.01	0.51 ± 0.02**	0.41 ± 0.03
σ_PSII_	643 ± 3	742 ± 16	591 ± 7	621 ± 3
P_max_ CO_2_-assimilation	0.030 ± 0.004	0.035 ± 0.005	0.032 ± 0.009	0.028 ± 0.009
P_max_ ETR_PSII_	174 ± 9*	330 ± 21	370 ± 26 *	506 ± 65
P_max_ Ф_e:C_ / n_PSII_	5874 ± 648*	9225 ± 1502	11691 ± 3730	18145 ± 6091
NPQ_NSV_	0.37–0.47***	0.58–0.75	0.5–0.59***	0.72–0.79

* p-value< 0.05

** p-value< 0.01

***p-value< 0.0001

*Thalassiosira oceanica* and *Chrysochromulina polylepis* were grown in steady state iron-replete and iron-limited conditions. The mean growth rate μ, derived from successive measurements in semi-continuous batch cultures, is given in d^-1^. The error is the SD of 3 biological replicates, and number of consecutive batch transfers (ca. 4 cell divisions per transfer) used to calculate growth rates are given in brackets. F_v_/F_m_ and σ_PSII_ are values from cultures in the dark regulated state (10 min of 5 μmol quanta m^-2^s^-1^ at 730 nm), measured on the day of CO_2_-assimilation experiments. The error is SD of 3 biological replicates. Changes in these parameters are statistically significant for *T*. *oceanica* (p-value < 0.0001) and *C*. *polylepis* (p-value < 0.01). P_max_ for CO_2_-assimilation (mol C mol chl*a* ^-1^ s^-1^) and ETR_RCII_ (mol e- mol RCII^-1^ s^-1^) were derived from P*vs*E curves as described in the methods section. The error is the 95% confidence interval of the P_max_ derived from the fit to data from 6 whole curve measurements (duplicate curves each from 3 biological replicates). The conversion factor Φ_e:C_/n_PSII_ for P_max_ was derived as the quotient of P_max_ for ETR_RCII_ and P_max_ for CO_2_-assimilation. The error is the propagated error from numerator and denominator. NPQ_NSV_ was estimated as F_o_′/F_v_′ from the last ST acquisition during each light level of the P*vs*E curves. The values shown are from the first and last step of the P*vs*E curves (4 and 800 μmol quanta m^-2^ s^-1^). Each NPQ_NSV_ value is the mean of 2 values measured on 3 biological replicates. Changes in response to iron limitation are statistically significant for both species (p-value < 0.0001).

## Discussion

Our results provide new insight into the effects of iron and light availability on the coupling between CO_2_-assimilation and photosynthetic electron transport in natural phytoplankton assemblages and mono-specific laboratory cultures. We show that both of these environmental variables significantly influence Φ_e:C_/n_PSII_, which has important implications for the use of FRRF measurements to infer rates of CO_2_-assimilation in oceanic waters. Below, we first discuss the observed increase in Φ_e:C_/n_PSII_ under excess light and low iron conditions in the context of previously reported values. We then discuss the effects of iron and light on phytoplankton photophysiology, and suggest a simple conceptual explanation for the observed increase in ETR_RCII_ under iron limitation. We hypothesize, that iron and light-dependent changes in Φ_e:C_/n_PSII_ are driven by the need to dissipate excess excitation energy, caused by either excess light, or the effects of iron limitation on the ETC. In this context, we discuss the correlation between Φ_e:C_/n_PSII_ and NPQ_NSV_, and examine the potential significance of this finding in the context of marine primary productivity studies.

### Magnitude of the observed conversion factor

The conversion factor Φ_e:C_/n_PSII_, derived from our measurements of ETR_RCII_ and CO_2_-assimilation, varied significantly in response to light and iron availability. In our field experiment, the addition of iron caused the value of Φ_e:C_/n_PSII_ at light saturation (P_max_) to decrease by 66% within 6 days (Fig 4F). Furthermore, short-term changes in light availability had a major effect on the value of Φ_e:C_/n_PSII_, and this effect was enhanced under iron limitation. A recent meta-analysis of variability in experimentally determined Φ_e:C_ from 14 field studies found values ranging from 1.15 to 54.2 with a mean of 10.9 ± 6.91 mol e^-^ mol C^-1^ [24]. This analysis comprised a wide range of oceanic regions, but did not include observations from the NE subarctic Pacific or other HNLC regions. Due to our experimental approach, we are unable to derive absolute values for Φ_e:C_. However, if we assume 1/n_PSII_ to be 500 mol chl*a* mol RCII^-1^ [20], as has been done in most previous studies [24], Φ_e:C_ values on day 3 of the iron-addition experiment range from 13 to 39 mol e^-^ mol C^-1^. Using a constant value of 1/n_PSII_ for both treatments is unlikely to be realistic. Even though iron-limited phytoplankton possess less chl *a* per cell, 1/n_PSII_, the ratio of chl *a* to RCII, has frequently been observed to increase under low iron conditions [84,85,88,90,98]. If we thus assume 700 mol chl *a* mol RCII^-1^ for the iron-limited control treatment and 500 mol chl *a* mol RCII^-1^ for the iron addition treatment [85], Φ_e:C_ ranges from 13 to 28 mol e^-^ mol C^-1^. These Φ_e:C_ values represent the range observed across different irradiance levels in our P*vs*E experiments. At the time of sampling, cells in the on board incubator were exposed to ~ 40 µmol quanta m^-2^ s^-1^. Assuming 700 and 500 mol chl *a* mol RCII^-1^ for the iron-limited and iron-replete treatments, respectively, we derive Φ_e:C_ values of ~18 and ~15 mol e^-^ mol C^-1^. Values of Φ_e:C_ estimated from our data are thus within the range reported in previous field studies [24], with no estimate falling below the theoretical minimum of 4 mol e^-^ mol C^-1^.

Ideally, measurements of ETR_RCII_ and CO_2_-assimilation should be performed simultaneously on the same sample, eliminating differences in incubation time and spectral quality of the light sources used. As discussed in detail in the supplementary material, the differences in spectral distribution of the light sources used for FRRF and ^14^C measurements could have led to an underestimation of absolute values of Φ_e:C_/n_PSII_ (S1 Fig). However, these differences cannot explain the large iron dependent changes we observed in Φ_e:C_/n_PSII_, since the absorption spectra of iron-limited and iron-enriched treatments did not differ drastically (S1 Fig). Furthermore, differences in incubation times could have influence the absolute magnitude of the derived conversion factor. Incubation times used for the P*vs*E curves were ca. 5 min for FRRF measurements (applied incrementally to the same sample), vs. 3–4 hours in the field and 30 min in the laboratory for ^14^C-assimilation experiments (light levels applied simultaneously to different samples). As has been shown by Halsey et al. [16,17,99] and Pei and Laws [18], the use of fixed incubation times for cells growing at different growth rates could lead to an overestimation of our conversion factor Φ_e:C_/n_PSII_ in the iron-limited relative to iron-replete samples. Additionally, the longer incubation time in CO_2_-assimilation experiments might have exacerbated cumulative processes such as photodamage under excess irradiance. To address this issue, we did not utilize the part of the P*vs*E curves showing photo-inhibition. However, we cannot rule out any differential cumulative effects of photoinhibition on ETR_RCII_ and ^14^C-assimilation at P_max_. This could potentially decrease CO_2_-assimlation at P_max_ relative to ETR_RCII_ at P_max_ and lead to overestimation of our Φ_e:C_/n_PSII_ values at P_max_. Notwithstanding these potential sources of uncertainty in the absolute value of Φ_e:C_/n_PSII_, the good agreement between our estimated Φ_e:C_ (assuming ~ 500–700 mol chl *a* mol RCII^-1^) and those of previous studies suggests that our observations are robust. More importantly, potential offsets in the absolute values of Φ_e:C_/n_PSII_ do not diminish the significance of the relative, iron and light-dependent changes we observed in this parameter (discussed below).

### Interacting effects of iron and light on the conversion factor Φ_e:C_/n_PSII_

Our data show strong and interacting effects of iron and light availability on the conversion factor Φ_e:C_/n_PSII_ in phytoplankton field assemblages and mono-specific laboratory cultures (Fig 4C, 4f and 6,Table 1). It has been shown that the magnitude of both 1/n_PSII_ and Φ_e:C_ vary significantly between phytoplankton taxa (e.g. [22,93]). Changes in Φ_e:C_/n_PSII_ in field experiments was thus likely influenced by both, physiological changes and taxonomic shifts. These two sources of variability are, to a large extent, intrinsically linked, since changes in phytoplankton community composition (S2 Fig) reflect the selection of better adapted species under any particular set of environmental conditions (i.e. iron limitation). In the following, we discuss the observed changes in Φ_e:C_/n_PSII_ from a predominantly photophysiological point of view, since our laboratory results specifically demonstrate such physiological effects.

Numerous metabolic processes, acting between ETR_RCII_ and CO_2_-assimilation can act to increase Φ_e:C_, and therefore the conversion factor Φ_e:C_/n_PSII_ (e.g. [61,59,100]). In addition to its role in reducing CO_2_ to organic carbon products, reductant (NADPH) formed at the end of the ETC can also be used for nitrate and sulphate reduction [101], photorespiration [102], or respiration via the malate shunt [103]. These alternative pathways decouple ETR_RCII_ from CO_2_-assimilation, increasing the value of Φ_e:C_. Similarly, before the formation of NADPH, pseudo-cyclic electron flow can reduce O_2_ and create a water-water cycle of electron transport, also increasing Φ_e:C_ (e.g. [104]). Pseudo-cyclic electron transport pathways can divert electrons from the ETC before (short water-water cycling, e.g. [105]) or after PSI (Mehler-reaction, e.g. [106]). Cyclic electron transport (CET) around PSII [107,108] and charge recombination in PSII [109,110], act more closely to the initial charge separation in RCII, and can also cause an increase in Φ_e:C_.

**Fig 8:**
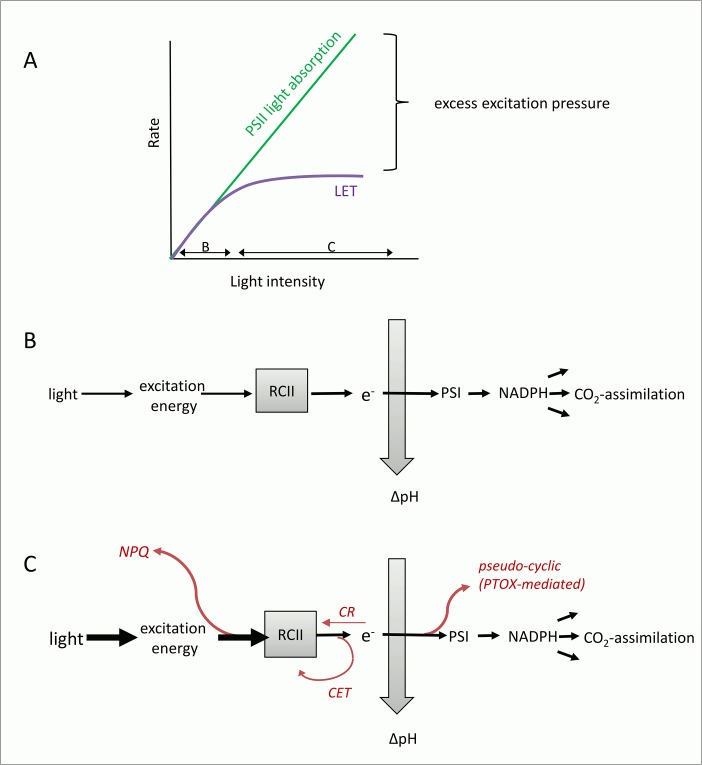
Conceptual diagram visualizing the concept of excess excitation pressure and its dissipation before and after charge separation in RCII. (A) Absorption of light energy by pigments in the light harvesting antenna of PSII cannot be controlled biologically, and rises linearly with incident light intensity. However, rates of linear electron transport (LET) and CO_2_-assimilation saturate at a light intensity determined by the physiological state of the phytoplankton, resulting in a typical P*vs*E curve. Under optimal growth conditions, it is the resupply of NADP^**-**^(predominantly from CO_2_-assimilation) which limits LET, while under short-term exposure to excess light and under iron limitation, the ‘bottleneck’ of LET will be located before PSI. Whenever exitonic influx exceeds the chemical outflux at the level of RCII, excess excitation pressure needs to be safely dissipated to prevent photodamage. (B) Under optimal growth conditions and sub-saturating light, all absorbed photons are used for charge separation in RCII, and the majority of electrons will be used for LET and CO_2_-assimilation, resulting in minimum Φ_e:C_. (C) Conditions of high excitation pressure can be caused by short-term exposure to high light, but also by iron limitation, which comprises the functioning of the ETC and has been shown to create a ‘bottle neck’ for LET before PSI. Under these conditions, PTOX-mediated pseudo-cyclic electron flow (e.g. [54–58,62,105,113]), cyclic electron transport around PSII (e.g. [107,108,114]), and charge recombination in RCII (e.g. [109,110,115]), have been suggested to safely dissipate excess excitation energy after RCII (but before PSI). Up-regulation of these alternative electron flow pathways could explain the high ETR_RCII_ (and Φ_e:C_/n_PSII_) observed in our iron-limited samples. Excess excitation energy can also be dissipated in the light harvesting antenna, before charge separation in RCII. Collectively, a number of different molecular processes dissipating excess excitation energy in the PSII antenna can be quantified as NPQ_NSV_.

We suggest that the higher Φ_e:C_/n_PSII_ observed in response to iron limitation and short-term increases in incident irradiance during the P*vs*E experiments (Fig 6A–6E) results predominantly from increases in the alternative electron flow pathways prior to reductant formation. These pathways, which are diagramed conceptually in Fig 8, can act as ‘safety valves’ to keep the primary quinone acceptor Q_A_ oxidized when excitation pressure on the ETC is high, thereby decreasing the potential of damage to RCII [111,54,55,61,56,104,112,60].

Iron limitation directly affects the photosynthetic ETC and thereby modulates the light-dependent changes in the conversion factor Φ_e:C_/n_PSII_ (Fig 6A–6E). Importantly, iron limitation has been shown to alter the stoichiometry of ETC components (i.e. expression of iron-rich PSI and cytochrome *b*_*6*_ *f* complexes is down-regulated to a higher extent than PSII) (e.g. [62,84,116–118]). Low levels of electron acceptors downstream of PSII ultimately restrict the flow of electrons away from PSII during light exposure. This exacerbates the need for short (i.e. acting before PSI) alternative electron flow pathways to dissipate excess excitation energy and prevent over-reduction of RCII (Fig 8). A number of recent studies have suggested that re-routing electrons to a midstream plastoquinol oxidase (PTOX) to bypass the electron flow bottleneck of PSI is a common strategy in open ocean phytoplankton [113,55,54,56–58,62]. Importantly, up-regulation of pseudo-cyclic electron flow under iron limitation not only protects RCIIs from photodamage, but also helps to maintain a high ΔpH across the thylakoid membrane, providing energy for cell maintenance and growth [62,119]. Cyclic electron flow around PSII [107,108,114,61,120] and increases in charge recombination at PSII [109,110,115] are two additional mechanisms that can act to prevent over-reduction and damage of RCII when excitation pressure is high and the electron flow bottleneck is prior to PSI. Unlike PTOX-mediated water-water cycling, these processes do not contribute to an increase in ΔpH across the thylakoid membrane. They would, however, contribute to a high ETR_RCII_ and therefore Φ_e:C_/n_PSII_ (Fig 8)_._

While ambient light intensity has a well-documented effect on values of 1/n_PSII_, these changes act on timescales longer than those of short-term P*vs*E experiments, and are thus unlikely to have caused the light-dependent changes we observed in Φ_e:C_/n_PSII_ (Fig 6). On longer time-scales, however, iron limitation causes a reduction of chl *a* per cell (chlorosis), and an increase in chl *a* per functional RCII (1/n_PSII_) [87,90]. This well documented response, which has been attributed to preferential down-regulation of RCII [87], and up-regulation of iron-stress-induced light harvesting complexes (isiLHCs) [62,90], would act to further increase Φ_e:C_/n_PSII_ under iron limitation, regardless of light intensity (Fig 6A–6E).

In summary, we suggest that it is the effect of high excitation pressure, which causes a de-coupling of ETR_RCII_ and CO_2_-assimilation. This high excitation pressure may be a result of short-term exposure to excess irradiance as well as the effect of iron limitation on the ETC. This purely photophysiological interpretation can be extended to observations made in mixed phytoplankton communities. Here, fluctuating light and low iron conditions will select for species with the best ability to control high excitation pressure by adjusting the flow of excitation energy into, and the flow of electrons out of PSII.

### Iron limitation increases ETR_RCII_

To our knowledge, this is the first study which shows that ETR_RCII_ increases under iron limitation. This observation may seem counter-intuitive, and it is important to emphasize that our results do not imply an overall increase in photosynthetic electron transport under low iron conditions. Rather, our observations point to an increase in the rate of charge separation at each individual RCII, independent of the reduced total cellular concentration of these RCII. We show that the overall efficiency of PSII photochemistry in the light-regulated state, F_q_′/F_m_′ (= Ф_PSII_′), is reduced under iron limitation (Fig 5C), as expected. However, deconvolution of this parameter into its constituents F_q_′/F_v_′ (Fig 5A) and F_v_′/F_m_′ (Fig 5B) shows that F_q_′/F_v_′, representing the fraction of open RCII (Q_A_ oxidized) at each given light level, increased under iron limitation. We hypothesize that this is likely achieved by increased alternative electron transport pathways acting to keep RCIIs open (Q_A_ oxidized) and bypassing the electron flow bottleneck at PSI, when excitation pressure is high (Fig 8). In contrast to F_q_′/F_v_′, the parameter F_v_′/F_m_′ is much lower when iron is limiting (Fig 5B), indicating that the excitation energy transfer in the antennae is compromised.

Based on our experimental observations, we suggest a simple mechanistic explanation for the observed increase in ETR_RCII_ under iron limitation. Cellular iron demand can be significantly reduced by economizing on iron-rich components of the photosynthetic apparatus and ‘funneling’ more electrons down fewer RCIIs (i.e., increasing ETR_RCII_). In line with this explanation is the observation that values of σ_PSII_ are high under iron limitation, and rapidly decrease after iron addition (Fig 2) [121,122,84,85,123–126,87]. Strzepek et al. [127] suggested that increased σ_PSII_ compensates for fewer iron-rich photosynthetic reaction centers in Southern Ocean phytoplankton species. Similarly, Ryan-Keogh et al. [128] noted that increasing the absorption cross section of RCs by the expression of isiLHCs allows cells to reduce the cellular iron requirement while maintaining the same light absorption capacity.

In conclusion, our results and interpretation support a scenario where photosynthetic electron flow has been fine-tuned to maximize energy conversion as well as photo-protection under conditions where ETC component abundance and stoichiometry are compromised by the availability of iron.

### Link to NPQ_NSV_

Above, we discussed how mechanisms acting down-stream of the initial charge separation in RCII are likely to be enhanced under conditions of high excitation pressure, resulting in high ETR_RCII_ and Φ_e:C_/n_PSII_. High excitation pressure can also be dissipated in the pigment antenna, before reaching RCII [104]. Fig 8 shows schematically the ‘safety mechanisms’ used for the dissipation of excess energy at both sides of RCII. Because processes dissipating excess excitation pressure in the antenna also quench ChlF yields measured by FRRF, they have collectively been called non-photochemical quenching (NPQ). NPQ, which is present in all oxygenic photosynthetic organisms, encompasses a wide variety of mechanisms acting to dissipate absorbed light energy as heat before it reaches RCII [129–134]. Following the approach of McKew et al.[80], we estimated NPQ from FRRF measurements as so-called normalized Stern-Volmer quenching (NPQ_NSV_). We observed a strong correlation between the conversion factor Φ_e:C_/n_PSII_ and the expression of NPQ_NSV_ (Fig 7). We note that Φ_e:C_/n_PSII_ and NPQ_NSV_ are not entirely independent parameters, and therefore the strong correlation observed in Fig 7 is in part a result of their co-dependence on the ChlF parameter F_v_′ (which we used in the derivation of both NPQ_NSV_ and Φ_e:C_/n_PSII_).

At this point, the relationship between Ф_e:C_/n_PSII_ and NPQ_NSV_ shown in Fig 7 is empirical rather than mechanistic. However, while there are a number of processes which will influence Ф_e:C_/n_PSII_ and NPQ_NSV_ differentially, there are many processes related to the amount of excitation pressure experienced by the ETC that would influence both in a consistent manner. Numerous studies have shown that Ф_e:C_ increases if light is saturating, i.e. when excitation pressure is high (e.g. [33,36,40]). Clearly, excess light would also increase the expression of NPQ_NSV_. Indeed, very recent work has pointed to a mechanistic link between alternative electron sinks involving PTOX and the expression of NPQ_NSV_ [105].

### A possible approach towards improved prediction of CO_2_-assimilation from FRRF data

While it remains to be seen how strong the correlation between Φ_e:C_/n_PSII_ and NPQ_NSV_ (Fig 7) may be for other datasets, our results provide a potential basis for improved estimates of CO_2_-assimilation from FRRF measurements alone. A number of factors make this approach more desirable than the use of static, regional conversion factors. First, the magnitude of Φ_e:C_/n_PSII_ in phytoplankton assemblages will be determined by a multitude of interacting environmental variables. The use of NPQ_NSV_ as an integrated physiological measure of environmental effects on electron transport processes will therefore help to constrain the relationship between Φ_e:C_/n_PSII_ and various environmental stressors. Secondly, as our data show, the magnitude of Φ_e:C_/n_PSII_ can vary significantly within the same sample in response to short-term variations in incident light. Such small scale changes would be lost using a static (regional) conversion factor, but are captured with our NPQ_NSV_-based approach, as every single ETR_RCII_ estimate is paired with a corresponding NPQ_NSV_ estimate. Finally, a non-static conversion factor is crucial if the goal is to monitor the effects of environmental change on marine primary productivity, since physiological responses to environmental change will likely affect the conversion factor itself before productivity changes are observed.

**Fig 9:**
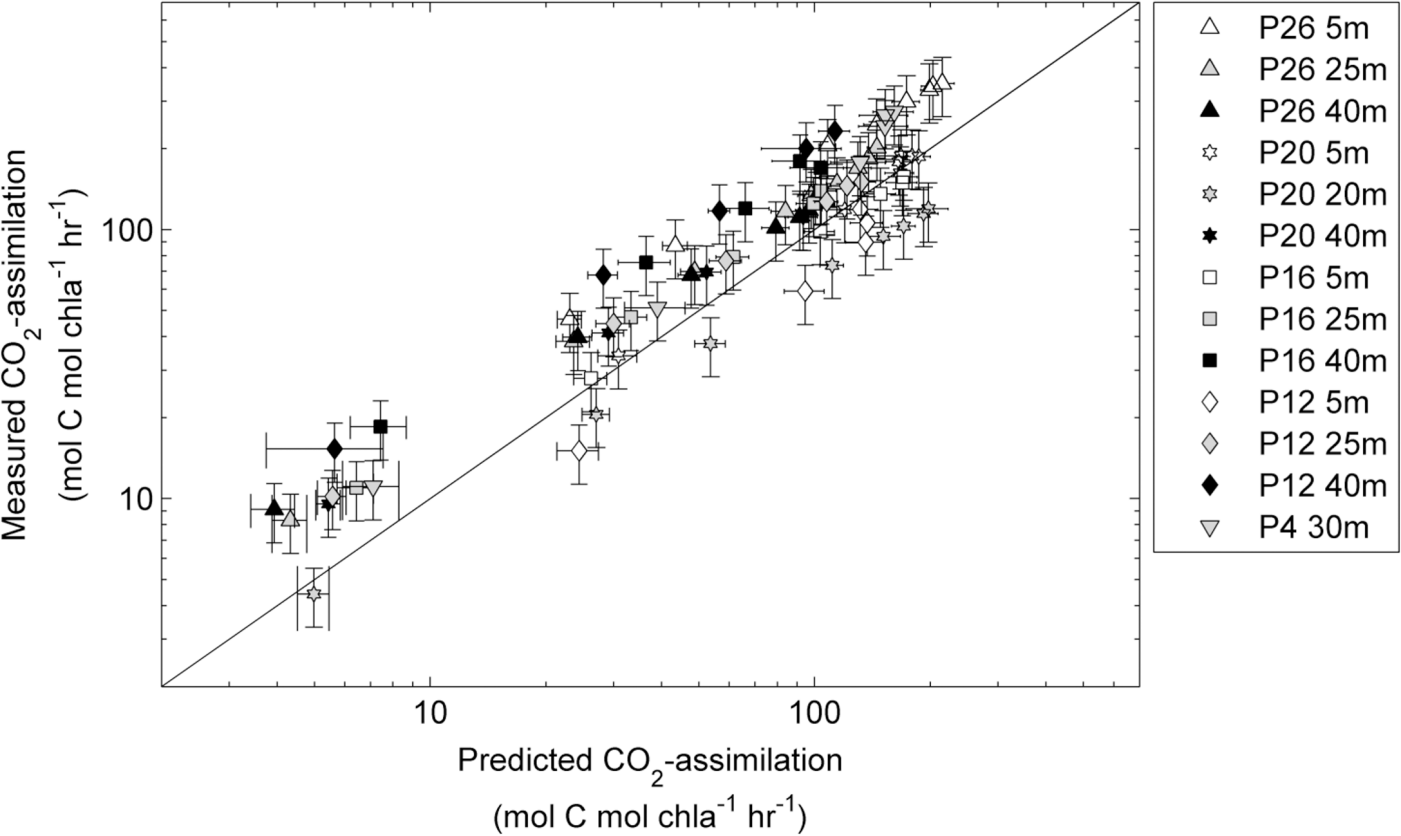
Rates of CO_2_-assimilation (mol C mol chl *a* ^-1^ hr^-1^) derived from FRRF measurements plotted against rates measured by ^14^C-assimilation experiments. Samples were taken at one to three depths at five stations along Line-P in the NE subarctic Pacific (see Fig 1). FRRF based P*vs*E curves were used to derive ETR_RCII_ and NPQ_NSV_ at 8 light levels for each sample, and Φ_e:C_/n_PSII_ values were then derived from the relationship presented in Fig 7. Φ_e:C_/n_PSII_ and ETR_RCII_ for each light level were used to calculate CO_2_-assimilation rates. Error bars for predicted CO_2_-assimilation rates represent the propagated error from the ChlF yields of the last three ST acquisitions of each light level during the FRRF P*vs*E curve used to derive NPQ_NSV_ and ETR_RCII_. Error bars for measured CO_2_-assimilation rates represent the mean coefficient of variance derived from all duplicate measurements (n = 46). The correlation between all predicted and measured data points (n = 95) was statistically significant (Spearman’s *r* = 0.90, two-tailed p-value < 0.0001). All statistics are for non log-transformed data.

As a test of the validity of our approach, we used the Φ_e:C_/n_PSII_ *vs*. NPQ_NSV_ correlation determined from our iron addition experiment (Fig 7) to predict the CO_2_-assimilation rates from FRRF-derived ETR_RCII_ and NPQ_NSV_ measured along the Line-P transect. In this case, in situ phytoplankton assemblages were collected from within and below the mixed layer, and rate measurements were conducted immediately after collection, without any experimental manipulation (see methods). As shown in Fig 9, we obtained a strong correlation between the predicted and measured CO_2_-assimilation rates (Spearman’ s *r* = 0.90, n = 95 and two-tailed p-value < 0.0001 on non log-transformed data). Our approach consistently underestimates values from the deepest sampling depth, which can likely be attributed to the lack of spectral correction of our data. The RMSE for the values predicted using our approach and measured values is 48.4 mol C mol chl *a* ^-1^ hr^-1^. This error represents ~ 10% of the total range of values observed along the transect during this study, suggesting that rates of productivity can be predicted with reasonable accuracy. In comparison with our approach, computation of CO_2_-assimilationfrom FRRF data assuming a constant 1/n_PSII_ value of 500 mol chl *a* mol RCII^-1^and 4 mol e^-^ mol C^-1^, significantly under-predicts observed CO_2_-assimilation rates (RMSE = 837.3 mol C mol chl *a* ^-1^ hr^-1^). Even if we use a constant conversion factor derived from the average of the Φ_e:C_/n_PSII_ measured during our iron addition experiment, the model error remains larger compared to that derived using our variable, NPQ_NSV_-based conversion factor (Fig 7). Our data therefore show significant potential in the application of a variable, NPQ_NSV_-derived conversion factor and associated quantification of carbon uptake rates from FRRF data.

## Conclusion

Deriving rates of phytoplankton CO_2_-assimilation from bio-optical approaches like FRRF has the potential to provide estimates of primary production at unprecedented spatial and temporal resolution. High resolution measurements, covering large oceanic regions, are essential for the monitoring and modelling of marine food webs and global biogeochemical cycles. Further, such measurements are indispensable for the development and validation of algorithms estimating global marine primary productivity from remote sensing.

Crucial to this approach is a sound characterization of the conversion factor between FRRF-derived ETR_RCII_ and primary productivity in carbon units. Our data demonstrate that the conversion factor varies significantly in response to iron and light availability in phytoplankton field assemblages and mono-specific laboratory cultures. We interpret the observed variability in the conversion factor Φ_e:C_/n_PSII_ as a manifestation of the extreme photophysiological flexibility which evolved in phytoplankton to maximize growth under dynamic light and nutrient regimes [135,136]. We hypothesize that, to a large extent, changes in Φ_e:C_/n_PSII_ represent a suite of coordinated photophysiological adjustments acting to balance light absorption with CO_2_-assimilation under given environmental conditions. These will be manifested on the physiological as well as on the taxonomic level. On the taxonomic level, a low nutrient and / or fluctuating light environment will select for species with the best ability to control high excitation pressure by adjusting the flow of excitation energy into, and the flow of electrons out of PSII (manifested in changes of NPQ_NSV_, 1/n_PSII_ and Φ_e:C_). Future studies will be needed to evaluate the relationship between NPQ_NSV_ and Φ_e:C_/n_PSII_ in a number of oceanic regions in order to evaluate the potential for improved CO_2_-assimilation estimates from FRRF data.

## Supporting Information

S1 Fig Spectral distribution of light sources used for FRRF and Photosynthetron assays, and absorption spectra of phytoplankton assemblages on day 6 of the iron-addition experiment. (a) The FRRF instrument used during this study contains LEDs with peak output at four wavelengths (445 nm, 470 nm, 503 nm, 530 nm). In our FRRF instrument, excitation as well as actinic background irradiance is applied from the same LEDs. (b) Spectral distribution of the LEDs used in the photosynthetron used for^14^C-uptake experiments. (c) Spectral overlap of the two light sources. The overlap is good in the region of maximal light absorption by photosynthetic pigment (ca. 450 nm). However, in direct comparison with the photosynthetron, the FRRF instrument provides a higher proportion of photons in the region > 480 nm. This could have led to an underestimation of ETR_RCII_ values relative to CO_2_-assimilation values measured in the photosynthetron, resulting in an under-estimate of Φ_e:C_/n_PSII_. In addition to knowledge of spectral differences in the light sources used (a-c), spectral correction of our data would require light absorption spectra of the phytoplankton assemblages examined. Relative absorption spectra of the phytoplankton communities on day 6 after iron-addition (measured using the quantitative filter technique [65]) are shown in (d-f). Spectra from 3 biological replicates of the control (d) and two biological replicates of the iron addition treatment (e) were averaged, and these spectra are shown together in panel (f). The results show relatively small changes in the relative light absorption between the two treatments, and it is unlikely that these changes would have significantly influenced the large iron and light-dependent effects in Φ_e:C_/n_PSII_. Because we did not measure absorption spectra for all sampling points of the iron addition experiment and stations along the transect, we were unable to spectrally correct our data. Furthermore, because we are not deriving absolute values for Φ_e:C_/n_PSII_, we did not apply a constant correction factor (estimated from e.g. the data shown in a-f).
(TIFF)

S2 Fig Phytoplankton assemblage composition on day 6 of the iron addition experiment. The taxonomic composition of phytoplankton assemblages (% of total chl *a*) was derived from HPLC analysis of accessory photosynthetic pigment. Average values are shown from three biological replicates for the iron-limited control and the iron addition treatment on day 6 of the experiment. One to1.5 L of water were filtered on 25 mm GF/F and stored at -80°C until analysis. Pigments were extracted and quantified as described by Taylor et al. [137]. Pigment ratios were then used to estimate phytoplankton assemblage composition using CHEMTAX as described by Taylor et al. [137]. The initial pigment ratio matrix used for our data was taken from Lee et al. [138], table 5, which is specific to North Pacific phytoplankton isolates.
(TIFF)

S3 Fig Response of volume normalized rates of CO_2_-assimilation (mol C m^-3^ hr^-1^) during the iron addition experiment. The rates were measured as a function of irradiance, and P*vs*E curves were fit with the exponential model of Webb et al. [74]. Shown are mean values from three biological replicates where error bars represent standard error of mean and are sometimes smaller than symbols. Results shown in this figure confirm a strong stimulatory effect of iron additions on primary productivity in the experimental bottles.
(TIFF)
